# A Preliminary Link between Hydroxylated Metabolites of Polychlorinated Biphenyls and Free Thyroxin in Humans

**DOI:** 10.3390/ijerph13040421

**Published:** 2016-04-13

**Authors:** Eveline Dirinck, Alin C. Dirtu, Govindan Malarvannan, Adrian Covaci, Philippe G. Jorens, Luc F. Van Gaal

**Affiliations:** 1 Department of Endocrinology, Diabetology and Metabolism, Antwerp University Hospital, University of Antwerp, Wilrijkstraat 10, Edegem 2650, Belgium; luc.van.gaal@uza.be; 2 Toxicology Centre, University of Antwerp, Wilrijk 2610, Belgium; alindirtu@yahoo.com (A.C.D.); malarvannan.govindan@uantwerpen.be (G.M.); adrian.covaci@uantwerpen.be (A.C.); 3 Department of Clinical Pharmacology, Antwerp University Hospital, University of Antwerp, Edegem 2650, Belgium; philippe.jorens@uza.be

**Keywords:** polychlorinated biphenyls, hydroxylated polychlorinated biphenyls, thyroid function, human, endocrine disrupters

## Abstract

*Background*: Polychlorinated biphenyls (PCBs) and their hydroxylated metabolites (HO-PCBs) interfere with thyroid hormone action both *in vitro* and *in vivo*. However, epidemiologic studies on the link between PCB exposure and thyroid function have yielded discordant results, while very few data are available for HO-PCBs. *Objectives*: Our study aimed at investigating the relationship between clinically available markers of thyroid metabolism and serum levels of both PCBs and HO-PCBs. *Subjects and Methods*: In a group of 180 subjects, thyroid-stimulating hormone (TSH) and free thyroxin (fT4), 29 PCBs (expressed both in lipid weight and in wet weight) and 18 HO-PCBs were measured in serum. *Results*: In regression models, adjusted for gender, age, current smoking behavior, BMI and total lipid levels, serum levels of 3HO-PCB118 and 3HO-PCB180, and PCB95_lw_, PCB99_lw_ and PCB149_lw_ were independent, significant predictors of fT4. A stepwise, multiple regression with gender, age, current smoking behavior, BMI and total lipid levels and all five previously identified significant compounds retained age, BMI, PCB95_lw_, PCB99_lw_ and 3HO-PCB180 as significant predictors of fT4. TSH levels were not predicted by serum levels of any of the PCBs or HO-PCBs. *Conclusions*: Our study indicates that *in vivo*, circulating fT4 levels can be linked to serum levels of several PCBs and hydroxylated PCB metabolites.

## 1. Introduction

The endocrine system plays an essential and pervasive role in the regulation of whole body homeostasis. The thyroid system is a classic neuro-endocrine axis, comprising a feedback system between the hypothalamus releasing thyrotropin-releasing hormone (TRH), the pituitary gland releasing thyroid-stimulating hormone (TSH) and the thyroid gland producing and secreting T4 and, to a lesser extent, T3. In the circulation, both hormones are protein bound (in humans to a high-affinity protein called TBG or thyroxine binding globulin) and less than 1% of these hormones circulate as free-T3 (fT3) and free-T4 (fT4). The feedback among the hypothalamus, pituitary, and thyroid maintains blood levels of thyroid hormones within relatively narrow limits [1]. Thyroid hormones are key players in the control system of our metabolism, with, amongst others, a pivotal role in energy metabolism, heart rate, and emotional stability [2,3]. 

Thyroid function can be influenced and disrupted by both endogenous and exogenous factors. Some of these factors are not amendable. TSH tends to increase during the lifespan [4,5], and is influenced by gender [6]. Other elements are more modifiable. Several reports have linked active smoking to lower TSH levels [7,8,9], whereas the influence of BMI is not as uniform [10,11]. Finally, lipid levels may also be associated with TSH [12].

Several man-made chemicals are suspected to have thyroid-disrupting properties [13,14,15]. Amongst them are the polychlorinated biphenyls (PCBs), a group of organochlorine chemicals. Since they are very resistant to extreme temperature and pressure, they were widely used in capacitors, transformers, hydraulic fluids, lubricants and as plasticizers. More than 200 individual PCB congeners have been identified in commercial mixtures, whose chemical and toxicological properties are related to the number and position of the chlorine atoms. Despite the ban on their production in Europe since the 1970s, their ongoing use, chemical stability, resistance to degradation, and lipophilicity has lead to significant bioaccumulation in most compartments of the ecosystem and human tissues [16,17]. This bioaccumulation leads to an ongoing human exposure to PCBs through a variety of pathways, dietary intake being the most important [18]. 

PCBs are slowly metabolized by the human body [19]. The first phase of this biotransformation process is mediated by cytochrome P450 monooxygenase isozymes, resulting in hydroxylated PCB compounds (OH-PCBs), which are persistent too and are slowly eliminated [20]. Many OH-PCBs are retained in the blood bound to proteins and, as such, have the potential to exert toxic effects [20].

Given their structural resemblance to thyroxin [21], both PCBs and OH-PCBs are under scrutiny for their potential role in thyroid disruption. In animal studies, both circulating free thyroxin (fT4), as well as serum triiodothyronine (fT3) were significantly reduced after exposure to PCB mixtures or individual congeners [22,23,24,25]. Some animal studies report that serum thyroid-stimulating hormone (TSH) is elevated by PCBs in response to low fT4 [26], whereas others report essentially no effect of PCB exposure on serum TSH [27,28].

Several studies have examined associations between PCB serum levels and thyroid hormones in humans. However, studies focusing on the link between hydroxylated PCB metabolites and thyroid function are scarce [29]. At present, taken into consideration the past and current exposure level in a Western population, thyroid function is considered a potential target for endocrine toxicity by persistent chemicals such as PCBs [30]. Our study aimed at investigating the relationship between serum levels of PCBs and, in particular, hydroxylated PCB metabolites, with clinically available markers of thyroid function in a group of normal weight, overweight and obese adults. 

## 2. Subjects and Methods 

### 2.1. Study Population 

Participants were prospectively recruited when visiting the Department of Endocrinology of the Antwerp University Hospital between November 2009 and February 2012. All participants were Belgian citizens, living in the vicinity of the Antwerp University Hospital, and all were sampled in the same time frame. Participants contacted the department due to existing obesity or diabetes. Participants were eligible if they were 18 years or older, and were overweight or obese. Participants were not eligible for the study if they were under 18 years of age, had type 1 diabetes mellitus, were pregnant or suffered from active psychiatric illness. Occupational exposure to PCBs was ruled out by detailed questioning by the physician. Patients with occupational exposure were excluded from the study. Since TSH values are influenced by age, gender and possibly BMI, a control group of age- and gender-matched normal weight individuals was recruited for this study. This study was approved by the local Ethical Committee (Belgian Registry number B30020097009) and registered at clinicaltrials.gov (number NCT01778868). All participants provided their written informed consent. A total of 196 participants were included in the study. Sixteen out of 196 participants (=8.2%) were excluded from further analyses, 15 participants due to the present use of thyroxin for hypothyroidism and one patient due to a significantly elevated TSH of 14.63 mU/L. Two male patients were diagnosed with subclinical hypothyroidism (TSH 4.37 and 5.05 mU/L respectively). We did not detect subclinical hyperthyroidism. All other patients (123 women and 57 men) were in a euthyroid state.

### 2.2. Thyroid Function 

Amongst all participants, history on thyroid disease was taken, and the use of current thyroid medication was recorded. Venous blood samples were obtained in the fasting state between 08.00 A.M. and 10.00 A.M. into sterile BD Vacutainer tubes. Thyroid Stimulating Hormone (TSH) and free Thyroxin (fT4) were measured using chemiluminescence Luminescence Oxygen Channeling Immunoassay (Dimension Vista, Siemens, Beersel, Belgium) at the hospital laboratory [31]. Reference values for TSH were 0.36–3.74 mU/L, and 9.8–18.8 pmol/L for fT4. We opted to focus on the clinically relevant markers TSH and fT4, as recommended by an expert panel [32].

### 2.3. Anthropometry 

Anthropometric measures were taken in the morning with individuals in a fasting state and undressed. Height was measured to the nearest 0.5 cm; body weight was measured with a digital scale to the nearest 0.2 kg. BMI was calculated as weight (expressed in kg), divided by the square of the height (expressed in meters).

### 2.4. Toxicological Analyses

Serum samples (typically 4.5 mL) were analyzed for 28 PCB congeners and 18 PCB metabolites according to the protocol described in detail by Dirtu *et al.* [33]. In short, solid-phase extraction (SPE) on OASIS HLB cartridges was used followed by fractionation on silica SPE cartridges topped with 0.5 g of acidified silicagel (44% H_2_SO_4_, *w/w*). The first fraction was eluted with *n*-hexane and contained neutral compounds, such as PCBs. The phenolic fraction eluted as second with dichloromethane and included HO-PCBs. The second fraction was derivatized with trimethylsilyl diazomethane (0.2 M in *n*-hexane) for 30 min at 60 °C. Both fractions evaporated to near dryness and reconstituted in iso-octane. The extracts were analyzed by gas chromatography coupled to mass spectrometry operated either in electron capture negative chemical ionization or electron impact mode depending on the sensitivity of the analytes. Further details on the analytical parameters and QA/QC protocols are given in Dirtu *et al.* [33].

PCB levels were expressed on a wet weight basis (PCB_ww_) and a lipid adjusted basis (PCB_lw_). All levels were added to create the sum of all PCBs (ΣPCBs). Total lipids were calculated using the formula: Total lipids (g/L) = total cholesterol (g/L) × 2.27 + triglycerides (g/L) + 0.62 [34]. Total cholesterol and triglycerides were measured at the Antwerp University Hospital, using a validated method (Siemens Dimension Vista 1500 System, Beersel, Belgium). Levels below the method level of quantification (LOQ) were assigned a value of DF × LOQ, with DF being the proportion (%) of measurements with levels above the LOQ or the detection frequency [35].

### 2.5. Statistical Analysis 

Statistical calculations were performed using SPSS, version 20.0 (SPSS Inc., Chicago, IL, USA). Normality of distribution was verified using the Kolmogorov-Smirnov test. All PCB and HO-PCB levels displayed a skewed distribution, which was transformable to normality after log transformation for most PCB and HO-PCB levels. TSH and fT4 were not normally distributed in our population, but were normalized after square root transformation and were used as such in the statistical analyses. Partial correlation analyses, using wet weight data and correcting for total lipids, were performed. To correct for multiple testing, a False Discovery Rate Correction Procedure was applied (with *q* = 0.05 and *m* = 47). Standard linear regression analyses were performed to assess the impact on fT4 and TSH levels. Residuals were normally distributed in all models. In the models, adjustments were made for gender, age, current smoking behavior (active *versus* non-smoker) and BMI, total lipids and a wet weight PCB serum level or PCB metabolite level. The analyses were repeated using a model containing gender, age, current smoking behavior, BMI and a lipid-adjusted PCB serum level. The same correction for multiple testing was applied (False Discovery Rate Correction Procedure with *q* = 0.05 and *m* = 47). After identification of significant contributors, these were inserted, together with gender, age, current smoking behavior, BMI and total lipids into a single forward stepwise regression analysis. A significance level of *p* < 0.05 was accepted for all analyses. 

## 3. Results

### 3.1. Study Population 

The study population consisted of 123 women (68%) and 57 men (32%) with a mean age of 41 ± 13 years (Table 1). The population selected consists of both lean and obese subjects. There was no statistically significant difference in age or sex between obese participants and lean participants. Analyses were not restricted to an obese population, given the data from the literature that suggest that obese patients might have a higher TSH level within the euthyroid range, compared to their lean counterparts. This observation is, however, not repeated in all studies [11]. In our population, there is no significant difference in TSH levels between obese and lean participants. Given most patients with type 2 diabetes are obese, several articles report a high frequency of elevated TSH in type 2 diabetes [36]. In the present study there is no statistical difference in TSH level between participants with type 2 diabetes, normal glucose tolerance or prediabetes (data not shown).

### 3.2. Toxicological Analysis

We detected all 28 PCB congeners and all 18 PCB metabolites in most of the subjects. Results on the exact levels are presented as supplementary information (Tables S1 and S2).

### 3.3. Partial Correlation Analysis 

After correction for multiple testing, no significant partial correlations between TSH or fT4 on the one hand and PCB_ww_ or HO-PCB serum levels on the other hand could be detected. 

### 3.4. Regression Analysis for fT4 

The models using PCB_lw_ serum levels revealed a significant contribution to fT4 levels for PCB95_lw_, PCB99_lw_ and PCB149_lw_ (Table 2, for a complete overview of all models, please see Supplementary Table S3). The models using a PCB metabolite serum level indicated a significant contribution to fT4 levels for 3HO-PCB118 and 3HO-PCB180 (Table 2, for a complete overview of all models please see Supplementay Table S4). Using the previously identified statistically significant PCB serum levels and hydroxylated PCB metabolite serum levels in a single forward stepwise procedure, PCB95_lw_, PCB99_lw_ and 3HO-PCB180 were identified as significant predictors of fT4 (Table 3). The wet weight PCB serum levels were not identified as significant predictors for fT4. 

### 3.5. Regression Analysis for TSH 

No PCB, either expressed as wet weight or lipid weight, or hydroxylated PCB metabolite could be identified as a significant predictor of TSH levels. 

## 4. Discussion

Polychlorinated biphenyls are suspected to disrupt endocrine balance, and in particular thyroid homeostasis. The mechanisms through which PCBs and their hydroxylated metabolites exert thyroid disruptive capacities have been unraveled in recent years, both *in vitro* and in animal studies [37]. PCBs are capable of interfering with thyroid hormone levels at many sites. At the DNA level, PCBs interfere with thyroid hormone receptor-mediated transcription [38]. Secondly, they affect synthesis associated proteins such as thyroid peroxidase [24]. *In vitro*, PCBs interfere with the sodium/iodide symporter [39] and compete with native hormone at the levels of transport protein [40,41] and act as agonists at the level of the thyroid receptor [42,43]. PCBs also enhance the excretion of thyroid hormones *in vitro* and in rats [22,26]. Finally, in fish, PCBs interfere with several iodothyronine deiodinases [44]. These interactions offer an clarification for the reduced fT4 levels, but the pathophysiological mechanism(s) causing blunting of a compensatory TSH rise remain largely unexplained. One possible mechanism has been documented in rats, indicating high doses of PCBs can induce impaired TRH-induced TSH release at the pituitary level [45,46]. As opposed to data on PCBs, data on the action mechanisms of PCB metabolites are scarce, and are largely derived from *in vitro* data with few *in vivo* data. Hydroxylated PCB metabolites disrupt thyroglobulin synthesis and excretion [47] and display a weak affinity for the human thyroid receptor [41,48]. *In vitro*, inactivation of thyroid hormones is inhibited by OH-PCBs due to limiting of the sulfotransferase activity [49]. Hydroxylated PCBs also interfere with thyroid hormone receptor-mediated transcription [50]. Increased T4 accumulation in the liver, inducing decreased serum T4, has been reported [51]. To date, no interference of PCB metabolites with TRH or TSH secretion has been documented. The lipid solubility of PCB metabolites is slightly lower than that of PCBs, which could explain the mechanistic differences. It should be noted that all of the studies mentioned above describe responses on acutely administered doses of PCBs and PCB metabolites. However, in the human situation, there is a chronic exposure, resulting from both low-level intake and internal exposure. It is unclear if the human body is capable of certain adaptive mechanism, for example by inducing CYP450 enzymes. 

Human studies have provided conflicting data on the exact nature of the resulting thyroid profile change. Our study aimed at investigating the relationship between serum levels of PCBs and, in particular, the hydroxylated PCB metabolites, with clinically available markers of thyroid function in an adult population. 

To our knowledge, we are the first to report 3HO-PCB118 and 3HO-PCB180 as independent, significant predictors of fT4. 3HO-PCB180 is a metabolite of PCB180 and PCB172, while 3HO-PCB118 can be formed from PCB118, PCB126 and PCB107. PCB118, PCB180 and PCB172 were also measured in our sample, but their levels did not show a significant link with fT4. We did not measure both other mother compounds of 3HO-PCB118. Dallaire reported a negative relation between serum levels of HO-PCB199 and HO-PCB208 and TSH levels in an adult Inuit population, but it should be noted that the concentration of hydroxylated PCB metabolites in their study was about a tenfold higher than in our sample [52]. In the same study, seven HO-PCBs were associated with decreased total T3 concentration [52]. Hagmar *et al.* measured 4HO-PCB107 and 4HO-PCB187 in a female cohort, yet they do not report on the link between these PCB metabolites and thyroid function [53]. In Vietnamese women, several PCBs and HO-PCBs correlated with fT4, and PCB74 and PCB118 correlated negatively with TSH; in Vietnamese men only PCB138 and PCB153 correlated significantly with fT4 and no relation with TSH could be detected [54]. Our findings underscore the need for further research into the endocrine disrupting capacities of hydroxylated PCB metabolites. 

When the different PCBs and PCB metabolites were analyzed individually, regression analysis indicates a significantly negative effect of PCB95_lw_ and PCB149_lw_, and a significantly positive effect of PCB99_lw_ on fT4 levels. Possibly as a consequence of these divergent influences, and the non-significant effect of most of the measured PCBs, the predictive effect of the sum of all PCBs was not significant in our population. The effect of PCBs on circulating levels of fT4 has been assessed by several previous studies. Free T4 was unaffected by levels of PCBs in several studies [53,55,56,57]. The study of Hagmar focused on PCB153, while all other studies analyze several PCB congeners. Concentrations of PCBs vary substantially between studies. In the studies focusing on participants with background exposure [52,58], concentrations of PCBs in our study are comparable. However, in studies focusing on participants with higher exposure levels, the PCB serum levels are up to an 8 fold higher compared to our study [53,55,56,57]. In the study of Langer *et al.*, fT4 levels were increased in participants with higher PCB levels [37]. However, it should be noted that the population studied by Langer *et al.* consisted of people living in a heavily polluted area. Their PCB levels were an order of magnitude higher than the levels detected in our cohort and only the effect of the sum of all examined PCBs was explored. In this perspective, it is of particular interest that the effect of individual PCB compounds diverged in our study. 

Our analyses indicate no effect of either PCBs or their hydroxylated metabolites on the TSH levels in our study sample. This is in line with the majority of the existing studies that did not detect a measurable influence on TSH levels [53,55,57,58,59,60,61,62,63]. The magnitude of PCB serum levels vary considerably between studies, as both groups with background exposure as groups with high exposure were studied. Most studies are limited to one gender [53,55,56,57,58,59,64]. It is somewhat puzzling that the remaining studies reported both increased and decreased TSH levels [56,62,64,65,66,67,68]. Four studies reported a decreased TSH with higher levels of PCBs, in 63 occupationally exposed men in the USA [56], in 89 women who miscarried in Germany [65], in 464 women and men in an industrially heavily polluted area of Slovakia [66] and in 545 fish consuming captains in the USA [67]. In a study by Turyk *et al*. [68] in 2445 American citizens with background exposure, influence on TSH levels was affected by age and gender, with a lower TSH in older men, and an elevated TSH in older women. Abdelouahab *et al.* detected no effect on TSH levels in Canadian women, while TSH levels were higher in fish consuming men with higher PCB levels [64]. 

When analyzing the different studies in humans, the difficulties in comparability are striking. First, the analytical techniques for PCBs and their metabolites differ, but most methods can accurately detect low levels. Secondly, often the sum of PCB congeners is used for analysis, but the number of measured PCB congeners differs between studies, as does the choice in International Union of Pure and Applied Chemistry (IUPAC) numbers. Thirdly, the analyzed media vary (serum, whole blood). Finally, some authors report wet weight data, while others use lipid-adjusted data. However, PCBs are capable of inducing dyslipidemia, even in the absence of obesity [69]. Disturbances in thyroid metabolism also give cause for lipid alterations. As such, using lipid-adjusted data to investigate the link between PCBs and thyroid metabolism may inadvertedly cause an overadjustment of reality. This caveat has recently been adverted by La Merril [70]. In this perspective, the significant influence of lipid adjusted PCB levels that were not reproducible with wet weight data, should be interpreted with caution. Hydroxylated PCB levels are, however, always reported as wet weight, due to their binding to proteins. As PCBs compete with thyroid hormone at the level of the transport protein *in vitro*, it is interesting to study this effect *in vivo* in men. However, since our study focused on TSH and fT4, without data on total T4 or total T3, this was not feasible. The available data on this topic show divergent result, with several studies reporting no effect on total T4 [53,55,56,59,61,64] or total T3 [55,56,60,62,64]. In Canadian and U.S. men with high PCB levels, total T4 is lower [58,62,64], a result that was replicated in a study with 51 U.S. women [62] and in a mixed gender Spanish study group [63]. 

Additionally, it should also be noted that *in vitro* and animal testing to study the effects of PCBs is often undertaken using a single PCB congener. However, the complexity of human exposure is extensive, given the vast array of compounds to which humans are exposed simultaneously. The concentrations of endocrine disrupting chemicals are often closely intercorrelated, providing additional difficulty in determining which of the compounds have an effect. In our study, HO-PCB180 was the only PCB metabolite to retain its statistical significance after stepwise multiple regression, while the components PCB95_lw_ and PCB99_lw_ also had statistical significance. As demonstrated by our results, grouping the levels of a certain class of chemicals might obscure the underlying opposing effects of individual compounds. 

As mentioned previously, most studies have not been able to detect a link between TSH levels and PCB serum levels. PCBs are capable of inducing an impaired TSH response [46], but it has not yet been explored if hydroxylated PCBs possess such capacities. However, from a clinical point of view, TSH elevation or reduction is considered to be the hallmark of impaired thyroid function. Even if the distinction between subclinical and overt thyroid disease is somewhat arbitrary because it depends to a considerable extent on the position of the patient’s normal set point for fT3 and fT4 within the laboratory reference range, subclinical hypo- and hyperthyroidism has been associated with increased, predominantly cardiovascular, morbidity [1,71]. Moreover, thyroid hormones are thought to exert a complex, though profound impact on several key components of the metabolic syndrome, such as weight control, blood pressure and glucose metabolism [72]. PCBs have been linked to the pandemic rise of obesity and associated diseases such as diabetes [73]. The possibility of an additional aggravating effect on these conditions through the thyroid system, certainly merits investigation. So although the effect of PCB and HO-PCB disturbance at the individual level might be small, the impact at population level, and its ensuing burden of disease and health care related costs, should be considered significant. 

This study has some limitations. Assessment of ongoing exposure through intake of items such as contaminated food or ingestion of dust has not been performed in this study population. PCB serum levels are known to increase slowly with age and acutely during weight loss [33]. As this study is cross-sectional, no information was available on past fluctuations in serum levels. If these fluctuations could have an effect on thyroid hormone metabolism, remains unclear. From the current knowledge, the authors cannot rule out that an obese and/or diabetic participant metabolizes PCBs into hydroxylated PCB metabolites differently than a lean and/or non-diabetic participant. Given the cross-sectional design of our study, a causal link between the measured levels of PCB and PCB metabolites and thyroid hormone levels cannot be established. Other chemicals, such as polychlorinated dibenzodioxins or polychlorinateddibenzofurans might also have the *in vitro* potency to disrupt thyroid metabolism, but were not studied in our population [13]. 

Our study suggests PCBs and their metabolites are associated with lower levels of fT4, without a simultaneous rise in TSH. Given the limited effects on fT4 and the lack of compensatory TSH rise, it remains uncertain if these changes are clinically relevant. In the future, a prospective, longitudinal study, focusing on TSH, fT4 and fT3 would help to unravel to what extend PCBs and their metabolites are capable of disrupting circulating fT4 and fT3 hormone levels and inhibiting the compensatory rise in TSH normally triggered by fT4 and fT4 decline. It would be interesting to investigate if such inappropriately low TSH is caused by an impaired TRH-response, or caused by a change in local fT4/fT3 signaling at the target tissues.

## 5. Conclusions

Our study indicates that *in vivo*, circulating fT4 levels can be linked to serum levels of several PCBs and hydroxylated PCB metabolites.These data add to the existing evidence that the endocrine disrupting capacities of PCBs and their metabolites can disrupt thyroid homeostasis. 

## Figures and Tables

**Table 1:** Clinical characteristics of 180 euthyroid study subjects.

Characteristics	No.	Percentage	Mean ± SD	Range
Gender				
Female	123	68		
Male	57	32		
Smoking				
Yes	28	16		
No	152	84		
Age	180		41 ± 13	18–84
BMI	180		35.1 ± 8.6	17.5–62.3
All subjects				
TSH (mU/L)	180		1.66 ± 0.80	0.34–5.05
fT4 (pmol/L)	180		13.5 ± 2.1	9.3–20.0
Female subjects	123			
TSH (mU/L) *	123		1.74 ± 0.77	0.40–3.58
fT4 (pmol/L)	123		13.5 ± 1.9	9.3–19.3
Male subjects	57			
TSH (mU/L) *	57		1.51 ± 0.84	0.34–5.05
fT4 (pmol/L)	57		13.4 ± 2.4	10.3–20.0

Legend: BMI = body mass index; * Significant difference (*p* < 0.05) between female and male subjects, as assessed by Mann-Witney U test.

**Table 2:** Linear regression analysis for fT4.

			Linear Regression Model		
POP	Significance	R^2^		β (95% CI)	*p*
PCB95_lw_	0.024	0.072			
			Gender	−0.024 (−0.113–0.066)	0.604
			Age	−0.001 (−0.004–0.003)	0.754
			BMI	0.007 (0.002–0.012)	0.004
			Smoking status	0.041 (−0.071–0.154)	0.470
			PCB95_lw_	−0.299 (−0.595–0.002)	0.048
PCB99_lw_	0.024	0.071			
			Gender	−0.026 (−0.116–0.064)	0.566
			Age	−0.003 (−0.007–0.001)	0.108
			BMI	0.006 (0.001–0.011)	0.012
			Smoking status	0.046 (−0.066–0.158)	0.421
			PCB99_lw_	0.143 (0.000–0.285)	0.049
PCB149_lw_	0.017	0.076			
			Gender	−0.030 (−0.121–0.062)	0.586
			Age	−0.001 (−0.005–0.004)	0.720
			BMI	0.006 (0.002–0.011)	0.009
			Smoking status	0.046 (−0.069–0.161)	0.505
			PCB149_lw_	−0.248 (−0.471–0.025)	0.030
3HO-PCB118	0.002	0.112			
			Gender	−0.024 (−0.113–0.065)	0.592
			Age	0.001 (−0.002–0.005)	0.442
			BMI	0.005 (0.000–0.010)	0.061
			Smoke	0.060 (−0.053–0.173)	0.293
			Total lipids	0.000 (−0.001–0.000)	0.024
			3HO-PCB118	−0.147 (−0.267–0.028)	0.016
3HO-PCB180	0.003	0.110			
			Gender	−0.041 (−0.130–0.049)	0.381
			Age	−0.002 (−0.007–0.002)	0.257
			BMI	0.010 (0.005–0.015)	0.000
			Smoke	0.083 (−0.030–0.196)	0.148
			Total lipids	0.000 (−0.001–0.000)	0.020
			3HO-PCB180	0.287 (0.047–0.527)	0.020

The model with an OH-PCB level included gender, age, BMI, current smoking behavior, total lipids and one serum HO-PCB level. The model with a PCB level included age, BMI, current smoking behavior and one serum PCB level (expressed as lipid adjusted in ng/g lipids); fT4 was used as a square root transformed variable. All HO-PCB and PCB levels were used after log transformation. Representation is limited to these models with a significant contribution of a PCB or HO-PCB. For details on all models, please check Supplementary.

**Table 3:** Forward stepwise regression analysis for fT4.

Variable	Β (95%CI)	*P*
Constant	3.563 (3.303–3.823)	<0.001
Gender	−0.015 (−0.100–0.071)	0.734
Age	−0.006 (−0.011–0.002)	0.007
Current smoking	−0.023 (−0.069–0.023)	0.321
BMI	0.007 (0.001–0.012)	0.013
PCB95_lw_	−0.412 (−0.763–0.061)	0.022
PCB99_lw_	0.309 (0.143–0.474)	<0.001
PCB149_lw_	−0.174 (−0.470–0.122)	0.248
3HO-PCB118	−0.104 (−0.233–0.025)	0.114
3HO-PCB180	0.251 (0.008–0.494)	0.043

The model included gender, age, current smoking behavior, BMI and total lipid levels, serum levels of 3HO-PCB118 and 3HO-PCB180, and PCB95_lw_, PCB99_lw_ and PCB149_lw_; fT4 was used as a square root transformed variable. All HO-PCB and PCB levels were used after log transformation.

## Supplementary Materials

The following are available online at www.mdpi.com/1660-4601/13/4/421/s1, Table S1: PCB levels in serum (*n* = 180), Table S2: PCB metabolite levels in serum (*n* = 180), Table S3: Linear regression for FT4 with lipid adjusted PCBs, Table S4: Linear regression for FT4 with hydroxylated PCB metabolites and total lipids.
