# Supplementary material for: A Preliminary Link between Hydroxylated Metabolites of Polychlorinated Biphenyls and Free Thyroxin in Humans
Source: Int J Environ Res Public Health. 2016 Apr 13;13(4):421. doi: 10.3390/ijerph13040421 (PMC4847083; doi:10.3390/ijerph13040421)
Supplement: Supplementary file 1 [file ijerph-13-00421-s001.pdf]

Supplementary Materials: A Preliminary Link between Hydroxylated Metabolites of Polychlorinated Biphenyls and Free Thyroxin in Humans

Eveline Dirinck, Alin C. Dirtu, Govindan Malarvannan, Adrian Covaci, Philippe G. Jorens and Luc F. Van Gaal

Table S1. PCB levels in serum (*n* = 180).

	Wet Weight Basis (ng/L)		Lipid Weight Basis (ng/g Lipid)	
PCB28	1	0–174	0.2	0–111
PCB52	0	0–20	0	0–20
PCB74	18	2–353	2.9	0.3–122
PCB95	1	1–14	0.2	0.1–5
PCB99	22	2–121	0.2	0.1–5
PCB101	1	0–17	0.2	0.1–6
PCB105	7	2–102	1.1	0.3–18
PCB118	41	4–448	6.9	0.7–165
PCB149	5	1–39	0.7	0.2–39
PCB146	26	2–163	4.2	0.3–65
PCB153	259	16–1262	43.1	2.5–624
PCB138	135	2–703	22.5	0.3–317
PCB187	37	2–327	6.2	0.4–95
PCB183	17	2–327	2.8	0.3–69
PCB128	0	0–42	0.1	0–42
PCB167	22	2–161	3.7	0.3–66
PCB174	2	0–9	0.4	0.0–7
PCB177	11	2–64	1.8	0.2–26
PCB171	6	2–33	1.0	0.2–17
PCB172	6	2–51	1.0	0.2–18
PCB156	23	2–217	3.8	0.3–71
PCB180	175	10–1136	27	1.6–432
PCB170	69	4–436	10 .5	0.7–167
PCB199	9	2–85	1.4	0.2–26
PCB196/203	15	2–82	2.3	0.2–40
PCB194	14	2–111	2.3	0.2–43
PBC206	2	1–14	0.4	0.1–7.0
PCB209	1	1–10	0.2	0.1–6.0

Results are expressed as median (minimum–maximum).

Table S2. PCB metabolite levels in serum (n = 180).

	Median	Range (min–max)
4–HO–PCB79	0.0	(0.0–52)
4–HO–PCB120	1.0	(0.3–5)
3–HO–PCB118	5.0	(0.9–49)
4–HO–PCB107	9.0	(1.0–77)
3–HO–PCB153	3.0	(0.7–23)
4–HO–PCB146	15.0	(1.0–131)
4–HO–PCB127	1.0	(0.3–11)
3–HO–PCB138	6.0	(0.8–51)
4–HO–PCB130	0.0	(0.0–2)
4–HO–PCB163	2.0	(0.7–12)
4–HO–PCB187	16.0	(1.0–81)
4–HO–PCB162	3.0	(0.9–17)
4–HO–PCB177	0.0	(0.0–3)
3–HO–PCB180	2.0	(0.6–19)
4–HO–PCB172	4.0	(0.8–29)
4–HO–PCB193	0.0	(0.0–4)
4–diHO–PCB202	0.0	(0.0–4)
4–HO–PCB208	0.0	(0.0–5)

Results are expressed in ng/L and represented as median (minimum–maximum).

Table S3. Linear regression for FT4 with lipid adjusted PCBs.

Linear Regression Model					
**POP**	**Significance**	**R^2**		**β (95% CI)**	***P***
PCB28	0.096	0.052			
			Gender	−0.027 (−0.118–0.063)	0.0552
			Age	−0.001 (−0.005–0.002)	0.491
			BMI	0.006 (0.002–0.011)	0.008
			Smoking status	0.053 (−0.061–0.168)	0.359
			PCB28	0.038 (−0.093–0.168)	0.568
PCB52	0.094	0.053			
			Gender	−0.030 (−0.121–0.061)	0.518
			Age	−0.001 (−0.004–0.002)	0.587
			BMI	0.006 (0.002–0.011)	0.008
			Smoking status	0.047 (−0.066–0.161)	0.412
			PCB52	−0.083 (−0.340–0.174)	0.525
PCB74	0.068	0.057			
			Gender	−0.038 (−0.114–0.068)	0.617
			Age	−0.116 (−0.007–0.002)	0.242
			BMI	0.193 (0.001–0.011)	0.011
			Smoking status	0.069 (−0.060–0.166)	0.356
			PCB74	0.107 (−0.060–0.218)	0.264

Table S3. *Cont.*

Linear Regression Model					
**POP**	**Significance**	**R^2**		**β (95% CI)**	***P***
PCB95	0.024	0.072			
			Gender	−0.024 (−0.113–0.066)	0.604
			Age	−0.001 (−0.004–0.003)	0.754
			BMI	0.007 (0.002–0.012)	0.004
			Smoking status	0.041 (−0.071–0.154)	0.470
			PCB95	−0.299 (−0.595–0.002)	0.048
PCB99	0.024	0.071			
			Gender	−0.026 (−0.116–0.064)	0.566
			Age	−0.003 (−0.007–0.001)	0.108
			BMI	0.006 (0.001–0.011)	0.012
			Smoking status	0.046 (−0.066–0.158)	0.421
			PCB99	0.143 (0.000–0.285)	0.049
PCB101	0.017	0.076			
			Gender	−0.014 (−0.104–0.077)	0.768
			Age	−0.001 (−0.004–0.003)	0.676
			BMI	0.006 (0.002–0.011)	0.009
			Smoking status	0.037 (−0.075–0.150)	0.516
			PCB101	−0.317 (−0.601–0.033)	0.029
PCB105	0.109	0.050			
			Gender	−0.028 (−0.119–0.063)	0.547
			Age	−0.001 (−0.005–0.003)	0.599
			BMI	0.006 (0.002–0.011)	0.008
			Smoking status	0.049 (−0.065–0.164)	0.396
			PCB105	0.002 (−0.204–0.208)	0.986
PCB118	0.105	0.050			
			Gender	−0.028 (−0.119–0.063)	0.547
			Age	−0.001 (−0.005–0.003)	0.599
			BMI	0.006 (0.002–0.011)	0.008
			Smoking status	0.049 (−0.065–0.164)	0.396
			PCB118	0.002 (−0.204–0.208)	0.986
PCB149	0.017	0.076			
			Gender	−0.030 (−0.121–0.062)	0.586
			Age	−0.001 (−0.005–0.004)	0.720
			BMI	0.006 (0.002–0.011)	0.009
			Smoking status	0.046 (−0.069–0.161)	0.505
			PCB149	−0.248 (−0.471–0.025)	0.030

Table S3. *Cont.*

Linear Regression Model					
**POP**	**Significance**	**R^2**		**β (95% CI)**	***P***
PCB146	0.109	0.050			
			Gender	−0.028 (−0.119–0.063)	0.542
			Age	−0.001 (−0.006–0.004)	0.653
			BMI	0.006 (0.002–0.011)	0.011
			Smoking status	0.050 (−0.064–0.163)	0.391
			PCB146	0.005 (−0.172–0.181)	0.958
PCB153	0.101	0.051			
			Gender	−0.028 (−0.118–0.063)	0.547
			Age	0.000 (−0.005–0.005)	0.948
			BMI	0.006 (0.001–0.011)	0.015
			Smoking status	0.048 (−0.066–0.162)	0.406
			PCB153	−0.034 (−0.186–0.118)	0.660
PCB138	0.089	0.053			
			Gender	−0.027 (−0.118–0.063)	0.550
			Age	0.000 (−0.004–0.005)	0.960
			BMI	0.006 (0.002–0.011)	0.009
			Smoking status	0.048 (−0.065–0.162)	0.401
			PCB138	−0.053 (−0.196–0.090)	0.468
PCB187	0.108	0.050			
			Gender	−0.028 (−0.119–0.063)	0.540
			Age	−0.001 (−0.006–0.004)	0.612
			BMI	0.007 (0.002–0.012)	0.010
			Smoking status	0.050 (−0.064–0.163)	0.389
			PCB187	0.011 (−0.153–0.176)	0.892
PCB183	0.109	0.050			
			Gender	−0.028 (−0.119–0.063)	0.544
			Age	−0.001 (−0.006–0.003)	0.638
			BMI	0.006 (0.002–0.011)	0.008
			Smoking status	0.049 (−0.064–0.163)	0.392
			PCB183	0.003 (−0.183–0.188)	0.975
PCB128	0.105	0.051			
			Gender	−0.028 (−0.118–0.063)	0.548
			Age	−0.001 (−0.004–0.002)	0.578
			BMI	0.006 (0.002–0.011)	0.008
			Smoking status	0.048 (−0.067–0.162)	0.412
			PCB128	−0.040 (−0.292–0.212)	0.754

Table S3. *Cont.*

Linear Regression Model					
**POP**	**Significance**	**R^2**		**β (95% CI)**	***P***
PCB167	0.104	0.051			
			Gender	−0.029 (−0.120–0.062)	0.526
			Age	0.000 (−0.005–0.004)	0.861
			BMI	0.006 (0.001–0.011)	0.016
			Smoking status	0.046 (−0.069–0.161)	0.434
			PCB167	−0.031 (−0.208–0.147)	0.733
PCB174	0.067	0.057			
			Gender	−0.026 (−0.116–0.064)	0.571
			Age	−0.001 (−0.004–0.002)	0.535
			BMI	0.006 (0.001–0.011)	0.015
			Smoking status	0.042 (−0.071–0.156)	0.463
			PCB174	−0.224 (−0.614–0.167)	0.260
PCB177	0.108	0.050			
			Gender	−0.028 (−0.119–0.062)	0.539
			Age	−0.001 (−0.006–0.003)	0.571
			BMI	0.007 (0.002–0.011)	0.008
			Smoking status	0.050 (−0.064–0.164)	0.388
			PCB177	0.016 (−0.182–0.213)	0.874
PCB171	0.103	0.051			
			Gender	−0.028 (−0.019–0.062)	0.540
			Age	−0.002 (−0.006–0.003)	0.484
			BMI	0.007 (0.002–0.011)	0.007
			Smoking status	0.051 (−0.063–0.165)	0.375
			PCB171	0.047 (−0.203–0.298)	0.711
PCB172	0.102	0.051			
			Gender	−0.029 (−0.120–0.062)	0.532
			Age	−0.002 (−0.006–0.003)	0.459
			BMI	0.007 (0.002–0.012)	0.008
			Smoking status	0.052 (−0.062–0.167)	0.368
			PCB172	0.051 (−0.180–0.182)	0.664
PCB156	0.109	0.050			
			Gender	−0.028 (−0.119–0.063)	0.541
			Age	−0.001 (−0.006–0.004)	0.645
			BMI	0.007 (0.001–0.012)	0.012
			Smoking status	0.050 (−0.064–0.164)	0.389
			PCB156	0.009 (−0.177–0.195)	0.926

Table S3. *Cont.*

Linear Regression Model					
**POP**	**Significance**	**R^2**		**β (95% CI)**	***P***
PCB180	0.106	0.051			
			Gender	−0.027 (−0.118–0.063)	0.552
			Age	0.000 (−0.006–0.005)	0.868
			BMI	0.006 (0.001–0.011)	0.020
			Smoking status	0.048 (−0.066–0.162)	0.406
			PCB180	−0.021 (−0.174–0.132)	0.785
PCB170	0.107	0.050			
			Gender	−0.028 (−0.118–0.063)	0.549
			Age	−0.001 (−0.006–0.005)	0.812
			BMI	0.006 (0.001–0.011)	0.016
			Smoking status	0.048 (−0.065–0.162)	0.402
			PCB170	−0.016 (−0.182–0.151)	0.851
PCB199	0.092	0.053			
			Gender	−0.029 (−0.120–0.062)	0.528
			Age	−0.002 (−0.007–0.002)	0.366
			BMI	0.007 (0.002–0.012)	0.006
			Smoking status	0.054 (−0.060–0.168)	0.351
			PCB199	0.071 (−0.134–0.275)	0.497
PCB196/203	0.107	0.051			
			Gender	−0.028 (−0.119–0.062)	0.539
			Age	−0.001 (−0.006–0.003)	0.580
			BMI	0.007 (0.002–0.012)	0.011
			Smoking status	0.051 (−0.064–0.165)	0.384
			PCB196/203	0.019 (−0.179–0.218)	0.847
PCB194	0.108	0.050			
			Gender	−0.028 (−0.119–0.063)	0.539
			Age	−0.001 (−0.006–0.004)	0.628
			BMI	0.007 (0.001–0.012)	0.014
			Smoking status	0.050 (−0.064–0.164)	0.389
			PCB194	0.010 (−0.187–0.208)	0.917
PCB206	0.106	0.051			
			Gender	−0.028 (−0.119–0.062)	0.538
			Age	−0.001 (−0.005–0.003)	0.509
			BMI	0.007 (0.002–0.011)	0.008
			Smoking status	0.052 (−0.063–0.167)	0.374
			PCB206	0.042 (−0.260–0.345)	0.785

Table S3. *Cont.*

Linear Regression Model					
**POP**	**Significance**	**R^2**		**β (95% CI)**	***P***
PCB209	0.107	0.051			
			Gender	−0.028 (−0.119–0.062)	0.538
			Age	−0.001 (−0.005–0.002)	0.524
			BMI	0.007 (0.002–0.011)	0.008
			Smoking status	0.051 (−0.064–0.165)	0.381
			PCB209	0.040 (−0.322–0.401)	0.829
SumPCB	0.107	0.051			
			Gender	−0.028 (−0.119–0.063)	0.545
			Age	−0.001 (−0.006–0.004)	0.805
			BMI	0.006 (0.001–0.011)	0.012
			Smoking status	0.048 (−0.065–0.162)	0.402
			SumPCB	−0.016 (−0.163–0.131)	0.829

The model included gender, age, BMI, current smoking behavior, and one serum PCB level (lipid adjusted); FT4 was used as a square root transformed variable; All PCB levels were used after log transformation.

Table S4. Linear regression for FT4 with hydroxylated PCB metabolites and total lipids.

Linear Regression Model					
**POP**	**Significance**	**R^2**		**β (95% CI)**	***P***
4HO-PCB79	0.023	0.082			
			Gender	−0.030 (−0.120–0.061)	0.519
			Age	0.001 (−0.003–0.004)	0.766
			BMI	0.008 (0.003–0.013)	0.002
			Smoke	0.077 (−0.039–0.193)	0.192
			Total lipids	0.000 (−0.001–0.000)	0.019
			4HO-PCB79	−0.028 (−0.135–0.078)	0.602
4HO-PCB120	0.022	0.083			
			Gender	−0.030 (−0.121–0.060)	0.507
			Age	0.001 (−0.003–0.005)	0.634
			BMI	0.007 (0.003–0.012)	0.002
			Smoke	0.072 (−0.042–0.186)	0.216
			Total lipids	0.000 (−0.001–0.000)	0.018
			4HO-PCB120	−0.121 (−0.507–0.266)	0.539
3HO-PCB118	0.002	0.112			
			Gender	−0.024 (−0.113–0.065)	0.592
			Age	0.001 (−0.002–0.005)	0.442
			BMI	0.005 (0.000–0.010)	0.061
			Smoke	0.060 (−0.053–0.173)	0.293
			Total lipids	0.000 (−0.001–0.000)	0.024
			3HO-PCB118	−0.147 (−0.267–0.028)	0.016

Table S4. *Cont.*

Linear Regression Model					
**POP**	**Significance**	**R^2**		**β (95% CI)**	***P***
4HO-PCB107	0.021	0.083			
			Gender	−0.029 (−0.120–0.061)	0.520
			Age	0.000 (−0.005–0.004)	0.852
			BMI	0.008 (0.003–0.013)	0.002
			Smoke	0.065 (−0.051–0.181)	0.268
			Total lipids	0.000 (−0.001–0.000)	0.024
			4HO-PCB107	0.060 (−0.109–0.229)	0.484
3HO-PCB153	0.017	0.086			
			Gender	−0.026 (−0.117–0.064)	0.567
			Age	−0.001 (−0.006–0.004)	0.593
			BMI	0.009 (0.003–0.015)	0.002
			Smoke	0.074 (−0.041–0.188)	0.205
			Total lipids	0.000 (−0.001–0.000)	0.027
			3HO-PCB153	0.0120 (−0.115–0.355)	0.315
4HO-PCB146	0.015	0.088			
			Gender	−0.030 (−0.120–0.060)	0.511
			Age	−0.002 (−0.007–0.004)	0.489
			BMI	0.009 (0.004–0.015)	0.001
			Smoke	0.072 (−0.042–0.186)	0.213
			Total lipids	0.000 (−0.001–0.000)	0.019
			4HO–PCB146	0.118 (−0.086–0.323)	0.255
4HO-PCB127	0.025	0.081			
			Gender	−0.031 (−0.121–0.060)	0.504
			Age	0.001 (−0.003–0.004)	0.740
			BMI	0.007 (0.002–0.012)	0.004
			Smoke	0.072 (−0.042–0.187)	0.216
			Total lipids	0.000 (−0.001–0.000)	0.018
			4HO-PCB127	−0.040 (−0.333–0.253)	0.787
3HO-PCB138	0.016	0.087			
			Gender	−0.025 (−0.116–0.065)	0.584
			Age	−0.001 (−0.007–0.004)	0.562
			BMI	0.009 (0.004–0.014)	0.001
			Smoke	0.074 (−0.041–0.188)	0.205
			Total lipids	0.000 (−0.001–0.000)	0.025
			3HO-PCB138	0.106 (−0.089–0.302)	0.285

Table S4. *Cont.*

Linear Regression Model					
**POP**	**Significance**	**R^2**		**β (95% CI)**	***P***
4HO-PCB130	0.025	0.081			
			Gender	−0.030 (−0.120–0.061)	0.519
			Age	0.000 (−0.003–0.004)	0.811
			BMI	0.008 (0.003–0.012)	0.002
			Smoke	0.072 (−0.042–0.187)	0.215
			Total lipids	0.000 (−0.001–0.000)	0.020
			4HO-PCB130	0.044 (−0.493–0.581)	0.871
4HO-PCB163	0.014	0.089			
			Gender	−0.032 (−0.122–0.058)	0.485
			Age	−0.002 (−0.006–0.003)	0.519
			BMI	0.009 (0.004–0.014)	0.001
			Smoke	0.072 (−0.042–0.186)	0.216
			Total lipids	0.000 (−0.001–0.000)	0.023
			4HO-PCB163	0.184 (−0.109–0.478)	0.217
4HO-PCB187	0.023	0.082			
			Gender	−0.032 (−0.123–0.059)	0.486
			Age	0.000 (−0.004–0.004)	0.935
			BMI	0.008 (0.003–0.014)	0.003
			Smoke	0.073 (−0.042–0.187)	0.212
			Total lipids	0.000 (−0.001–0.000)	0.018
			4HO-PCB187	0.057 (−0.159–0.272)	0.603
4HO-PCB162	0.024	0.082			
			Gender	−0.031 (−0.121– 0.059)	0.499
			Age	0.000 (−0.005–0.004)	0.942
			BMI	0.008 (0.003–0.013)	0.003
			Smoke	0.067 (−0.050–0.184)	0.262
			Total lipids	0.000 (−0.001–0.000)	0.023
			4HO-PCB162	0.056 (−0.199–0.312)	0.665
4HO-PCB177	0.022	0.083			
			Gender	−0.029 (−0.119–0.061)	0.528
			Age	0.000 (−0.004–0.004)	0.993
			BMI	0.008 (0.003–0.013)	0.002
			Smoke	0.073 (−0.041–0.188)	0.209
			Total lipids	0.000 (−0.001–0.000)	0.020
			4HO-PCB177	0.091 (−0.199–0.381)	0.536

Table S4. *Cont.*

Linear Regression Model					
**POP**	**Significance**	**R^2**		**β (95% CI)**	***P***
3HO-PCB180	0.003	0.110			
			Gender	−0.041 (−0.130–0.049)	0.381
			Age	−0.002 (−0.007–0.002)	0.257
			BMI	0.010 (0.005–0.015)	0.000
			Smoke	0.083 (−0.030–0.196)	0.148
			Total lipids	0.000 (−0.001–0.000)	0.020
			3HO-PCB180	0.287 (0.047–0.527)	0.020
4HO-PCB172	0.010	0.094			
			Gender	−0.037 (−0.127–0.053)	0.420
			Age	−0.003 (−0.008–0.003)	0.334
			BMI	0.010 (0.004–0.016)	0.001
			Smoke	0.073 (−0.040–0.187)	0.205
			Total lipids	0.000 (−0.001–0.000)	0.024
			4HO-PCB172	0.203 (−0.051–0.457)	0.116
4HO-PCB193	0.002	0.112			
			Gender	−0.034 (−0.123–0.055)	0.447
			Age	−0.001 (−0.005–0.003)	0.541
			BMI	0.009 (0.004–0.014)	0.000
			Smoke	0.071 (−0.042–0.184)	0.215
			Total lipids	0.000 (−0.001–0.000)	0.025
			4HO-PCB193	0.365 (0.071–0.658)	0.015
4diMeO-PCB202	0.025	0.081			
			Gender	−0.030 (−0.120–0.061)	0.517
			Age	0.001 (−0.003–0.004)	0.796
			BMI	0.008 (0.003–0.004)	0.002
			Smoke	0.072 (−0.043–0.187)	0.217
			Total lipids	0.000 (−0.001–0.000)	0.019
			4diMeO-PCB202	−0.007 (−0.330–0.315)	0.964
4HO-PCB208	0.020	0.084			
			Gender	−0.029 (−0.120–0.061)	0.521
			Age	0.000 (−0.003–0.004)	0.882
			BMI	0.008 (0.003–0.013)	0.002
			Smoke	0.073 (−0.042–0.187)	0.211
			Total lipids	0.000 (−0.001–0.000)	0.018
			4HO-PCB208	0.121 (−0.169–0.410)	0.412

Table S4. *Cont.*

Linear Regression Model					
**POP**	**Significance**	**R^2**		**β (95% CI)**	***P***
sum HO-PCBs	0.020	0.084			
			Gender	−0.031 (−0.121–0.059)	0.500
			Age	−0.001 (−0.006–0.004)	0.722
			BMI	0.009 (0.003–0.014)	0.003
			Smoke	0.070 (−0.045–0.184)	0.231
			Total lipids	0.000 (−0.001–0.000)	0.020
			Sum HO-PCBs	0.091 (−0.148–0.329)	0.455

© 2016 by the authors; licensee MDPI, Basel, Switzerland. This article is an open access article distributed under the terms and conditions of the Creative Commons by Attribution (CC-BY) license (http://creativecommons.org/licenses/by/4.0/).
